# The Development of a 3D PET Fibrous Scaffold Modified with an Umbilical Cord dECM for Liver Tissue Engineering

**DOI:** 10.3390/polym16131794

**Published:** 2024-06-25

**Authors:** Yang Li, Yang Zhang, Kebo Zhong, Shuguang Liao, Guifeng Zhang

**Affiliations:** 1 National Key Laboratory of Biochemical Engineering, Institute of Process Engineering, Chinese Academy of Sciences, Beijing 100190, China; liyang2343@163.com (Y.L.); zhangyang21@ipe.ac.cn (Y.Z.); 2 School of Chemical and Engineering, University of Chinese Academy of Sciences, Beijing 100049, China; 3 Institute of Regenerative Medicine, Zhujiang Hospital, Southern Medical University, Guangzhou 510280, China; zhongkb@smu.edu.cn (K.Z.); xiaoliao0803@126.com (S.L.)

**Keywords:** PET, decellularized extracellular matrix, tissue engineering, three-dimensional culture, scaffold, modification

## Abstract

Organ and tissue dysfunction represents a clinically significant condition. By integrating cell biology with materials science, tissue engineering enables the reconstruction and restoration of damaged tissues or organs, offering a noninvasive repair approach. In our study, we replicated the cellular growth environment by utilizing a human umbilical cord-derived decellularized extracellular matrix (dECM) as a modifying agent for the polyethylene terephthalate (PET) polymeric fiber scaffold. This allowed us to create a dECM-coated polyester fiber-based scaffold, PET-dECM, tailored for liver tissue engineering purposes. We effectively produced a decellularized human umbilical cord-derived ECM through a combined decellularization process involving trypsin/EDTA, TritonX-100, and sodium deoxycholate. The application of the dECM coating onto the PET material was accomplished through several steps, such as ester hydrolysis, EDC/NHS-activated crosslinking, and dECM conjugation. The biological performance of the PET-dECM was validated using RG cell culture assays. Notably, the dECM coating significantly improved PET’s hydrophilicity and biocompatibility, thereby aiding cell adhesion, proliferation, and functional differentiation (*p* < 0.05). It was further found that the hepatocyte function of HepaRG was significantly enhanced on the PET-dECM, which may be attributed to the dECM’s ability to facilitate the restoration of cell polarity. The PET-dECM holds promise as an effective hepatocyte culture carrier and could potentially find application in liver tissue engineering.

## 1. Introduction

The failure of organ and tissue functions is a leading cause of human health hazards and one of the primary reasons for patient mortality [1,2,3,4]. The liver, a crucial organ for life, is the largest substantive organ and gland in the human body, boasting a remarkably intricate and distinctive structure [5]. Its physiological functions include storing glycogen; participating in the metabolism of sugars, fats, proteins, vitamins, and hormones; detoxifying the body; secreting bile; synthesizing coagulation factors; regulating blood volume; and maintaining water and electrolyte balance. When various liver diseases lead to the massive necrosis of liver cells, it can induce liver failure, resulting in common complications such as jaundice, coagulation disorders, septicemia, renal dysfunction, and hepatic encephalopathy, ultimately threatening the patient’s life [6,7,8,9]. The clinical manifestations of liver failure are complex, with rapid progression, high mortality, and an extremely poor prognosis [10].

Liver transplantation remains the only effective treatment for liver failure. Despite being technically mature, its widespread clinical application is constrained by the limited supply of donor livers, meaning less than 10% of patients with liver failure or severe liver disease are able to receive timely liver transplantation [11,12,13]. The vast disparity between the number of patients awaiting liver transplantation and the availability of donor livers has compelled researchers to explore various treatment methods that can support patients safely through the perioperative period of transplantation. The emergence of liver tissue engineering has brought hope to patients with liver failure or severe liver disease.

The three fundamental elements of tissue engineering are seed cells, biomaterials, and tissue construction. Its basic philosophy lies in combining life science and engineering methods to construct artificial organs, thereby restoring or partially replacing the functions of damaged organs [14,15,16]. During the construction of artificial organs, physiological activities such as cell proliferation, differentiation, and metabolism are regulated and influenced by the microenvironment. Cells cultured in a two-dimensional setting exhibit significant differences from the three-dimensional environment within the body, leading to disparities in cell morphology, differentiation, cell–matrix interactions, and cell–cell interactions compared to their physiological behaviors *in vivo*. Inside the body, hepatocytes grow within a natural three-dimensional network scaffold composed of various extracellular matrices (ECMs). These ECMs form a complex network structure that not only supports and connects tissues but also regulates tissue formation and cellular physiological activities. A crucial principle in artificial organ construction is simulating the *in vivo* cellular environment to enhance the function of cells cultured *in vitro*. Consequently, a strategy in artificial organ construction involves creating a three-dimensional culture system that mimics the physiological environment *in vivo*, enabling cells to behave more closely to their actual physiological conditions in the body. As an effective attachment for adherent cells, scaffold materials directly impact the microenvironment for cell growth, determining cell behavior and fate [17,18,19]. By simulating the *in vivo* environment and preparing a three-dimensional porous network structure scaffold, it is possible to provide a highly simulated *in vitro* environment for a hepatocyte culture. The porous structure design of this scaffold offers several advantages: Firstly, it provides ample growth space and a broader attachment area for cells. Additionally, this design facilitates the smooth entry of nutrients into cells and the efficient removal of metabolic waste, effectively avoiding potential growth inhibition issues encountered during *in vitro* cell culture. This, in turn, enhances the cell culture density and metabolic activity [20]. By seeding hepatocytes into specific ECMs, these matrices serve as growth scaffolds, guiding cell differentiation and producing structures with three-dimensional tissue specificity. This approach maximizes the simulation of the *in vivo* environment, enabling hepatocytes to exhibit physiological characteristics and functions similar to those *in vivo*, even in an *in vitro* setting.

The extracellular matrix (ECM) is a complex secreted by the cells of tissues and organs, distributed on the cell surface and within intercellular spaces. It comprises an intricate mix of structural and functional proteins, including type I collagen, type III collagen, fibronectin, laminin, glycosaminoglycans (GAGs), and various cell growth factors [21,22]. Collagen has been proven as a tissue engineering material that significantly promotes cell adhesion, proliferation, and functional expression [23,24,25,26]. However, collagen is only one component of the ECM and cannot fully simulate the complex growth environment of hepatocytes. Decellularized extracellular matrix (dECM) materials, including decellularized tissues and organs, are obtained through a decellularization process that removes cells and other antigenic components, retaining the primary components of the natural ECM. These biomaterials, exhibiting excellent bioactivity, are designed as substitutes for biological activity. They are intended to restore or enhance the structural integrity and functional capabilities of damaged tissues or organs in the human body, and have found widespread applications in tissue regeneration and repair, covering the liver, urinary bladder, lung, heart valve, blood vessel, esophagus, cornea, and kidney [27,28,29]. Studies have utilized liver decellularized matrix hydrogels to cultivate hepatocytes, promoting hepatocyte growth and improving liver function *in vivo* [30,31]. Further research has revealed that the decellularized liver matrix-coated cryogel-based bioreactor for bioartificial liver (BAL) support exhibits improved liver functions in rodent liver failure models. Additionally, it can enhance liver failure conditions in patient-derived liver failure plasma by approximately 30–60% [32]. The Food and Drug Administration has approved the Urinary Bladder Matrix (UBM) and Small Intestinal Submucosa (SIS) as commonly used dECM biomaterials, which have demonstrated successful outcomes in the regeneration of damaged skin, muscle, and gastrointestinal tissues [33]. Umbilical cord (UC) tissue offers several advantages as a tissue engineering material: it is rich in ECMs, exhibits low immunogenicity, is easy to obtain and process, possesses biosafety, and enjoys higher ethical and regulatory acceptance. These strengths make umbilical cord tissue an ideal source of biomaterial [34,35,36]. In this study, a polyethylene terephthalate (PET) fibrous scaffold is employed as the base material. Through modification, activation, and crosslinking steps, the UC dECM is coated onto the PET fibrous scaffold’s surface. This approach aims to simulate the *in vivo* microenvironment, constructing a novel tissue engineering scaffold for large-scale hepatocyte culture and functional expression.

## 2. Materials and Methods

### 2.1. Preparation and Analysis of Umbilical Cord dECM

The study protocols were reviewed and approved by the review board and ethics committee of Zhujiang Hospital of Southern Medical University (approval number: 2022-KY-003-01). The umbilical cord was sourced from clinical waste, and informed consent was obtained from the mothers. Decellularization: The umbilical cord was cut into suitable thin slices and placed in ultrapure water for shaking and cleaning at room temperature. After being cleaned, the tissue was immersed in a 0.025% trypsin-EDTA solution (Thermo Fisher Scientific, Waltham, MA, USA) and shaken for 1.5 h in a water bath maintained at 37 °C. The trypsin-EDTA solution was then discarded, and the tissue was washed with deionized water. A 5% Triton X-100 solution (Macklin Inc., Shanghai, China) was added, and the mixture was shaken for another 1.5 h. Following this, the umbilical cord tissue was placed in a 4% sodium deoxycholate solution (Macklin Inc., Shanghai, China) and shaken for 2 h at 25 °C. The tissue was then thoroughly washed with deionized water to remove any reagent residues. Freeze-drying: A vacuum freeze-dryer (LGJ-20F, SONG YUAN FREEZE DRYER, Beijing, China) was used to freeze-dry the umbilical cord tissue for 48 h, obtaining a dehydrated, freeze-dried human umbilical cord decellularized matrix. Grinding: Finally, the freeze-dried matrix was cut and added to a grinding instrument (LUKYM-I, Luka, Guangzhou, China), where it was ground at 55 HZ for 180 s, ultimately resulting in a fine powder of a human umbilical cord decellularized matrix.

To evaluate the decellularized tissues, histological analyses and biochemical assays were performed. For the histological analyses, both native and decellularized umbilical cord tissues underwent fixation with 4% paraformaldehyde (Macklin Inc., Shanghai, China), followed by paraffin embedding. Subsequently, the tissue sections were prepared using a microtome (RM216, Leica, Bensheim, Germany). These tissue slides were stained with hematoxylin and eosin (H&E) (Beyotime Biotechnology, Shanghai, China), periodic acid-silver methenamine (PASM) (LEAGENE Biotechnology, Beijing, China), periodic acid-schiff (PAS) (Beyotime Biotechnology, Shanghai, China), and Masson’s trichrome (MT) (Beyotime Biotechnology, Shanghai, China) after deparaffinization. To further observe the specific location of the decellularized matrix components in the tissue sections, immunofluorescence staining was performed on these paraffin sections. These tissue slides were stained, respectively, with polyclonal antibodies against collagen I (Proteintech Group, Inc., Chicago, IL, USA), collagen II (Proteintech Group, Inc., Chicago, IL, USA), collagen IV (Proteintech Group, Inc., Chicago, IL, USA), elastin (Proteintech Group, Inc., Chicago, IL, USA), lamin (Proteintech Group, Inc., Chicago, IL, USA), and fibronectin (Proteintech Group, Inc., Chicago, IL, USA). After reacting with the secondary antibodies, 4′,6-diamidino-2-phenylindole (DAPI) (Beyotime Biotechnology, Shanghai, China) was added to stain the nuclei. The quantity of DNA present in the dECM was determined through genomic DNA extraction (Tiangen Biotech (Beijing) Co., Ltd., Beijing, China) and subsequently measured using a nanodrop micro-nucleic acid meter (Thermo Fisher Scientific, Waltham, MA, USA). The dsDNA content in the DNA extraction solution was converted to the dsDNA content per milligram of dry tissue powder weight. Scanning electron microscopy (SEM, JEOL JSM-6700F, Akishima, Japan) was utilized to observe and compare the morphological disparities between the native UC tissue and the decellularized UC tissue. Initially, the tissues underwent dehydration through a gradient ethanol wash for 15 min each step at room temperature. Subsequently, they were dried employing CO_2_ critical point drying (K850, Quorum, Brighton, Britain) and coated with platinum using a sputter coating (JFC1600, JEOL, Akishima, Japan). Afterward, the samples were observed and photographed using a JSM-6700F scanning electron microscope (JEOL, Akishima, Japan). According to the manufacturer’s instructions, a Sircol^TM^ Soluble Collagen Assay (Biocolor Life Sciences, Carrickfergus, UK), a Blyscan^TM^ sGAG assay kit (Biocolor Life Sciences, Carrickfergus, UK), and a Fastin^TM^ elastin Assay (QuickZime Biosciences, Leiden, The Netherlands) were used to determine the collagen, glycosaminoglycan (GAG), and elastin contents of the UC tissues, respectively.

### 2.2. Preparation and Characterization of PET Fibrous Scaffold Modified with Umbilical Cord dECM

#### 2.2.1. Preparation of Human UC dECM Hydrogel

The UC dECM powder, prepared according to the method described in Section 2.1, was weighed out and dissolved in a 1% pepsin solution (Thermo Fisher Scientific, Waltham, MA, USA) prepared with hydrochloric acid, until the powder reached a final concentration of 10 mg/mL in the solution. A magnetic stirrer was used to continuously stir the mixture for 2–3 days until the decellularized matrix powder was completely digested, resulting in a grayish-white, thick consistency.

#### 2.2.2. Pretreatment and NaOH Modification of PET Fibrous Scaffolds

The commercially available medical-grade PET fibrous scaffolds were sliced into uniformly sized sheets measuring 2.0 cm × 0.6 cm × 0.3 cm. Before the modification, the PET fibrous scaffolds underwent sequential washes with ethanol, acetone, and distilled water while being ultrasonicated. Then, they were treated with 5% NaOH (Tianjin Damao Chemical Reagents Factory, Tianjin, China) at 40 °C for 40 min to expose the carboxyl groups (PET-COO) by hydrolyzing the ester bonds, ultimately yielding PET-COO scaffolds.

#### 2.2.3. UC dECM Hydrogel Modification of Fibrous Scaffolds

The PET-COO fibrous scaffolds were soaked for 1 h in a 0.1 M MES solution (Beijing Xin Jing Ke Biotechnology Co., Ltd., Beijing, China) adjusted to a pH of 5.6. Subsequently, 2 mM of EDC (Tokyo Chemical Industry, Tokyo, Japan) and 6 mM of NHS (Shanghai Aladdin Biochemical Technology Co., Ltd., Shanghai, China) were added to the MES solution. This mixture was maintained at 25 °C for 30 min to facilitate further activation and crosslinking reactions, ultimately producing PETHS scaffolds. Meanwhile, the pH of the UC dECM hydrogel was adjusted to 7.2 and then diluted to 5 mg/mL with PBS. The scaffolds were then immersed in this hydrogel and incubated at 25 °C for 2 h to promote coupling. Following incubation, the scaffolds were washed and dried, yielding PET-dECM scaffolds.

#### 2.2.4. Characterization of PET-dECM Fibrous Scaffolds

The Fourier transform infrared (FTIR) spectra of the PET, PET-COO, and PET-dECM scaffolds were acquired using a NICOLET iS50 FT-IR spectrometer (Thermo Fisher Scientific, Waltham, MA, USA). The surface wettability of the PET, PET-COO, and PET-dECM was evaluated using a contact angle goniometer (K100, Kruss, Hamburg, Germany). A droplet volume of 2 μL was used, and the contact angles were measured at 0 s, 5 s, and 10 s after placing the water droplets on the surface of the samples. The composition and structure of the surface layer on the scaffolds were analyzed using an X-ray photoelectron spectroscope spectrometer (ESCALAB 250Xi, Thermo Fisher Scientific, Waltham, MA, USA). The C1s, N1s, and O1s spectra were collected over a binding energy range of 0–1350 eV with a step size of 1 eV. To analyze the components of the dECM coating on the scaffold samples, 10 mg of the samples was weighed out and denatured in 50 mM NH_4_HCO_3_ at 110 °C for 10 min. After cooling and drying, 20 mg of trypsin solution was added to the samples, which were then incubated at 37 °C for 18 h. Following enzymatic hydrolysis, the supernatant was separated through centrifugation and then concentrated to 100 uL. This concentrated sample was analyzed using high-performance liquid chromatography (UltiMate 3000 RS, Thermo Fisher Scientific, Waltham, MA, USA) coupled with mass spectrometry (TSQ QUANTUM ACCESS MAX, Thermo Fisher Scientific, Waltham, MA, USA) for the HPLC-MS analysis.

### 2.3. Cell Culture on the Scaffolds

#### 2.3.1. Culture of HepaRG Cells on the Scaffolds

The scaffolds, measuring (2.0 cm × 0.6 cm × 0.3 cm) and sterilized by electron beam radiation, were positioned in 12-well plates sourced from BIOFIL (Guangzhou, China). HepaRG cells were grown in William’s E Medium, fortified with Metabolism Medium Supplement, both sourced from Thermo Fisher Scientific (Waltham, MA, USA). These cells were cultivated at 37 °C in a CO_2_ incubator manufactured by SANYO (Osaka, Japan). The HepaRG cells in their logarithmic growth phase were converted into a cell suspension and seeded into the scaffolds at a concentration of 2 × 10^6^ cells/mL. The nutrient solution for the cells was renewed every second day.

#### 2.3.2. Observation of Cell Morphology

Following 1, 3, 6, and 9 days of cell cultivation, the morphological disparities between the HepaRG cells cultivated on the PET, PET-COO, and PET-dECM scaffolds were observed and compared through scanning electron microscopy (SEM, JEOL JSM-6700F, Akishima, Japan). Prior to observation, the scaffolds underwent a preparation process: they were initially rinsed three times with PBS and then preserved with 2.5% glutaraldehyde at 4 °C for a full night. Afterward, the scaffolds were dehydrated by exposure to a graded ethanol series, with each wash lasting 15 min at room temperature. The drying process involved the use of CO_2_ critical point drying (K850, Quorum, Brighton, Britain) and a platinum sputter coating (JFC1600, JEOL, Akishima, Japan). Subsequently, the prepared samples were examined and photographed with the aid of a JSM-6700F scanning electron microscope (JEOL, Akishima, Japan).

#### 2.3.3. Cell Proliferation Assay

To evaluate the vitality of the HepaRG cells grown on the PET, PET-COO, and PET-dECM scaffolds, a Live/Dead Cell Staining kit (KeyGEN BioTECH, Nanjing, China) was used. After discarding the supernatant and washing the samples with PBS, a staining mixture of calcein (AM) and propidium iodide (PI) was applied. The cells were incubated for 30 min and then examined under a fluorescence microscope (Axio Vert A1, ZEISS, Oberkochen, Germany) after applying fluorescent mounting media.

To determine the survival rate of the HepaRG cells cultivated on the PET, PET-COO, and PET-dECM scaffolds, the supernatant was discarded and the samples were rinsed with PBS and then incubated with lysis buffer (Reagent A100, Chemometec, Allerod, Danmark) containing 0.04% trypan blue (leagene, Beijing, China) to liberate and stain the cell nuclei. The blood counting chamber was employed to tally the stained nuclei.

The cell proliferation was assessed utilizing the Cell Counting Kit-8 (CCK-8, sourced from Biosharp, Hefei, China) across three replicates. After discarding the culture medium, the samples were rinsed thrice with PBS. Then, 150 µL of CCK-8 solution was dispensed into each well, followed by incubation at 37 °C for a duration of 3 h. Post-incubation, 90 µL of the reacted solution was pipetted into a 96-well plate (BIOFIL, Guangzhou, China). The optical density (OD) of the samples was then determined at 450 nm with the aid of a microplate reader (Multiskan GO, Thermo Fisher Scientific, Waltham, MA, USA).

#### 2.3.4. Cell Function Assay

The detection of the albumin synthesis gene (ALB) and cytochrome P450 3A4 (CYP3A4) in the HepaRG cells was carried out with a Human ELISA kit (Jiangsu Meimian Industrial Co., Ltd., Yancheng, China). To assess the ALB, samples were collected from the culture supernatant at various time intervals (1, 3, 6, and 9 days). Meanwhile, for CYP3A4 detection, samples were derived from the cell lysate by employing a repetitive freeze–thaw technique. The optical density (OD) of each specimen was determined at 450 nm. To gauge the ammonia-to-urea conversion by the HepaRG cells cultivated on the PET, PET-COO, and PET-dECM scaffolds, the supernatant, collected at the aforementioned time points, was replaced with a complete medium enriched with 3 mM NH_4_Cl. Following a 90 min incubation in a carbon dioxide incubator, the medium was harvested for an analysis of the urea synthesis.

The total RNA was extracted from the HepaRG cells cultured on the PET, PET-COO, and PET-dECM scaffolds on day 6 using TRIzol reagent (Thermo Fisher Scientific, Waltham, MA, USA). For quantitative reverse-transcription polymerase chain reaction (qRT-PCR), the total RNA was reverse-transcribed to cDNA. Next, the cDNA was quantified using real-time PCR with SYBR Green qPCR master mix (Thermo Fisher Scientific, Waltham, MA, USA). The tested genes included polarity genes: ATP-binding cassette subfamily C member 2 (*ABCC2*), *Occludin*, and sodium-taurocholate cotransporting polypeptide (*NTCP*); stemness genes: sry-box transcription factor 9 (*SOX9*), leucine-rich repeat containing G protein coupled receptor 5 (*LGR5*), and recombinant octamer-binding transcription factor 4 (*OCT4*); and function genes: *ALB*, urea cycle gene (*CPS1*), and *CYP3A4*. *β-Actin* was used as a reference gene. The primers used in this study are listed in Table 1.

### 2.4. Statistical Analysis

All of the quantitative data were expressed as the mean ± standard deviation. The statistical significance between two groups was analyzed using Student’s *t*-test, and differences between three groups were determined using one-way ANOVA, followed by a Tukey HSD post hoc test. A *p*-value threshold of 0.05 was set for considering results as statistically significant.

## 3. Results and Discussion

### 3.1. Preparation and Analysis of Umbilical Cord dECM

**Figure 1:**
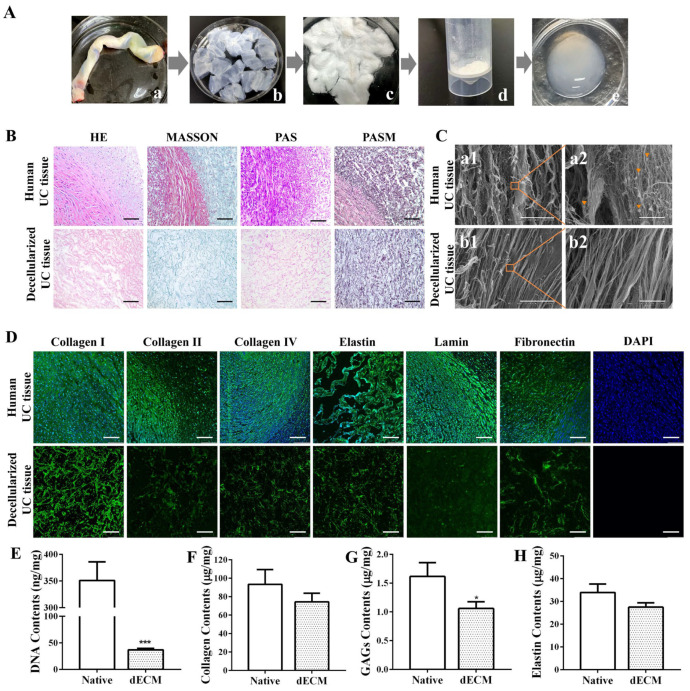
Preparation and analysis of umbilical cord decellularized extracellular matrix (dECM). (**A**) Preparation process of umbilical cord dECM. a: Umbilical cord tissue, b: umbilical cord decellularized matrix, c: freeze-dried umbilical cord decellularized matrix, d: umbilical cord decellularized matrix powder, e: umbilical cord acellular matrix gel. (**B**) Immunohistochemical staining. Scale bar: 100 μm. (**C**) SEM analysis. Scale bar: 200 μm (a1, b1); 10 μm (a2, b2). 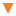: cell structure. (**D**) Immunofluorescence staining. Scale bar: 100 μm. (**E**) DNA content. Biochemical assays for (**F**) collagen, (**G**) glycosaminoglycans (GAGs), and (**H**) elastin in native UC and UC dECM. Data are given as mean ± SD, *n* = 3; *: *p* < 0.05, and ***: *p* < 0.001.

The preparation process of an umbilical cord dECM was demonstrated (Figure 1A). After undergoing a series of decellularization treatments, including trypsin and chemical detergents, the original umbilical cord tissue (Figure 1(Aa)) transitions to a transparent state (Figure 1(Ab)). Subsequently, additional lyophilization processing reveals a loose, sponge-like structure in the umbilical cord tissue (Figure 1(Ac)). Once these tissue blocks are crushed and pulverized, they are transformed into a milky white matrix powder (Figure 1(Ad)). Finally, after being digested with hydrochloric pepsin, the umbilical cord extracellular matrix gel manifests as an opaque white, sleek, and viscous colloidal material (Figure 1(Ae)). During the preparation process of the decellularized matrix, we have incorporated multiple methods, including the use of biological enzymes, physical stirring, and chemical reagents like anionic and cationic detergents, to achieve a more efficient and comprehensive decellularization effect [37,38,39].

The degree of decellularization was assessed using histological analyses (Figure 1B). The results of H&E staining showed numerous cellular structures visible in the untreated tissue, with the nuclei clearly stained blue, indicating an abundance of cellular components. However, after the decellularization, the tissue exhibited no obvious cellular remnants, and the extracellular matrix appeared bright red. Masson trichrome staining revealed that, in untreated fresh umbilical cord tissue, collagen fibers were stained blue, cytoplasm was stained red, and nuclei were stained purple-black. After decellularization, only blue was visible in the tissue, suggesting that the scaffold retained a significant amount of collagen fiber components, and most cells were removed. Both the PAS and PASM staining results demonstrated that the scaffold after decellularization showed no obvious nuclear staining, preserving a considerable amount of extracellular matrix components, including collagen and polysaccharides. These results confirm that cellular components were successfully removed, while key components of the decellularized tissues were effectively retained in the UC ECM following the decellularization process.

The ultrastructure of the umbilical cord decellularized extracellular matrix (dECM) was observed by scanning electron microscopy (Figure 1C). The results indicate that the surface structure of fresh umbilical cord tissue without decellularization (Figure 1(Ca1)) appears loose and disordered, possibly due to the presence of cells and their related structures. In contrast, the ECM tissue after elution treatment (Figure 1(Cb1)) exhibits smoother and flatter structural characteristics. Meanwhile, through enlarged photography, cell structures can be observed in the fresh umbilical cord tissue (Figure 1(Ca2)), while no obvious cell residues are found in the dECM group (Figure 1(Cb2)). The results suggest that the elution treatment effectively removes cellular components, allowing for the ultrastructure of ECM tissue to be clearly visible. Additionally, it can be observed that the decellularized tissue does not show obvious structural damage, further demonstrating the gentleness and effectiveness of this treatment method.

The immunohistochemical staining results of the umbilical cord tissue before and after decellularization were assessed using specific protein antibodies (Figure 1D). Compared to fresh umbilical cord tissue, the decellularized umbilical cord tissue exhibits varying degrees of expression in collagen, laminin, and fibronectin, stained in green. Among these proteins, the expression of type I collagen is particularly evident. This suggests that the decellularization technique effectively preserves laminin, elastin, fibronectin, type I collagen, type II collagen, and type IV collagen, thereby retaining most of the extracellular matrix components. Meanwhile, based on the results of the DAPI immunostaining, no obvious blue nuclear staining is observed under a fluorescence microscope. This finding further confirms the significant elution effect of this decellularization protocol on cells within the umbilical cord tissue, effectively removing cellular components while preserving the important extracellular matrix.

The quantification of the DNA content (Figure 1E) revealed that approximately 89.53% of the DNA was removed during the decellularization process, leaving only about 36.76 ± 2.22 ng/mg in the decellularized tissue [40]. Compared with the control group, there is a statistically significant difference (*p* < 0.001). Furthermore, the decellularized tissues retained 74.45 ± 7.57 μg/mg of collagen (Figure 1F), 1.06 ± 0.09 μg/mg of GAGs (Figure 1G), and 27.49 ± 1.59 μg/mg of elastin (Figure 1H). Compared to the natural umbilical cord tissue, the protein composition of the decellularized tissue decreased, with a statistically significant reduction observed in GAGs (*p* < 0.05).

### 3.2. Characterization of PET-dECM Fibrous Scaffolds

**Figure 2:**
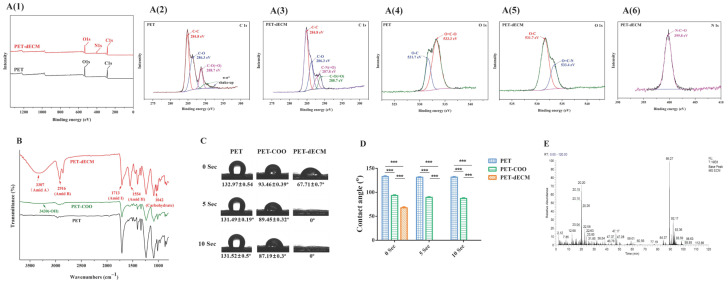
The characterization of the PET-dECM fibrous scaffolds. (**A**) The XPS spectra of the PET and PET-dECM scaffolds. The spectra include (A(1)) the overall survey, (A(2)) C1s for PET, (A(3)) C1s for PET-dECM, (A(4)) O1s for PET, (A(5)) O1s for PET-dECM, and (A(6)) N1s for PET-dECM. (**B**) FTIR spectra of PET, PET-COO, and PET-dECM scaffolds. (**C**) The water contact angles of the PET, PET-COO, and PET-dECM scaffolds at three time points (0 s, 5 s, and 10 s). (**D**) The statistical histogram of the water contact angle in the different samples. (**E**) The HPLC-MS analysis of the total ion chromatogram of the PET-dECM scaffold. The data are given as the mean ± SD, *n* = 3; ***: *p* < 0.001.

Polyethylene terephthalate (PET), chemically represented by the formula (C10H8O4)n, is a polymer made up of carbon, hydrogen, and oxygen atoms. The XPS spectra obtained for the PET and PET-dECM are presented in Figure 2(A(1–6)). Initially, in the full XPS spectrum of all the substrate surfaces, we detected signals corresponding approximately to binding energies of 284.8 eV and 531.7 eV, indicating the presence of C1s and O1s. After the PET was modified with the dECM, the XPS spectrum of the PET-dECM showed the emergence of a N1s signal at approximately 398 eV, suggesting the successful incorporation of nitrogen (N) (Figure 2(A(1))). Figure 2(A(2)) displays the C1s spectrum obtained for PET, with peaks at 284.8 eV, 286.3 eV, and 288.7 eV assigned to C-C, C-O, and C-O(=O), respectively. Meanwhile, due to the presence of conjugated π bonds in PET, there is a π-π* shake up at the high binding energy end. The binding energy peak at 287.8 eV in Figure 2(A(3)) corresponds to the C-N(=O) groups, confirming the successful dECM modification of the PET matrix. The O1s spectrum exhibits peaks at 533.3 eV (Figure 2(A(4))) and 533.4 eV (Figure 2(A(5))), assigned to O=C-O and O=C-N, respectively, indicating the occurrence of modification reactions. The N1s spectrum of PET-dECM shows binding energies at 399.8 eV (Figure 2(A(6))), corresponding to the amide groups formed after dECM modification. The peak assignments are consistent with those documented in previous studies, indicating the successful modification of PET via dECM treatment [24,41,42].

Additionally, the surface element analysis of the PET-dECM revealed that it retained 67.03% of the C element, 23.53% of the O element, and 8.95% of the N element (Table 2). Compared to PET fibers, the PET-dECM exhibited a decrease in the contents of the C and O elements and an increase in the content of the N element, suggesting that the surface element composition has changed after the modification treatment.

The PET material, as a representative polyester polymer, abundantly features ester groups within its molecular structure [43]. The ester group represents a distinctive chemical linkage resulting from the esterification reaction between a carboxyl group and an alcohol group. Upon exposure of PET material to an alkaline environment, the hydroxide ions (OH-) present in the medium actively engage in nucleophilic addition reactions with the carbonyl carbon (C=O) of the ester bond. This sequence of events initiates the cleavage of the ester bond, ultimately leading to its hydrolysis, yielding a carboxyl group (-COOH) and a hydroxyl group (-OH). To gain a deeper understanding of the fibrous surface modification process, we employed the FTIR technique. We observed that, in contrast to PET, PET-COO demonstrates a broadened absorption peak at 3430 cm^−1^ (Figure 2B), which is characteristic of hydroxyl groups, subsequent to the hydrolysis of ester groups. Through further EDC/NHS activation, crosslinking, and coupling with natural biomaterials derived from the extracellular matrix onto PET-COO, the PET-dECM displays characteristic absorption peaks of collagen at 1554 cm^−1^, 1713 cm^−1^, 2916 cm^−1^, and 3307 cm^−1^, which correspond to Amide II, Amide I, Amide B, and Amide A, respectively. Additionally, a characteristic absorption peak indicating the presence of carbohydrates is observed at 1042 cm^−1^ [44].

Figure 2C shows the results of the water contact angle measurements taken for the PET fiber-based scaffold materials, both before and after the dECM coating treatment, at various time points (0 s, 5 s, and 10 s). These findings offer a clear insight into how the treatment process affects the material’s hydrophilicity. The untreated PET material exhibited water contact angles exceeding 90° at all three time points, highlighting its relatively strong hydrophobic nature. However, substantial alterations were observed when the PET fiber-based scaffolds underwent NaOH modification followed by dECM coating [45]. Specifically, the water contact angles for PET-COO were recorded as 93.46 ± 0.39°, 89.45 ± 0.32°, and 87.19 ± 0.3° at 0 s, 5 s, and 10 s, respectively. On the contrary, the water contact angle for the PET-dECM dropped significantly to 67.71 ± 0.7° at 0 s and plummeted to 0° at both 5 s and 10 s. This outcome underscores that PET-dECM materials can be fully saturated with water in a remarkably short timeframe, showcasing remarkable hydrophilicity. When juxtaposed with the untreated PET, the alterations in the water contact angle among the materials treated with NaOH and coated with the dECM proved to be statistically significant (*p* < 0.001) (Figure 2D).

Figure 2E presents the total ion chromatogram (TIC) derived from the HPLC-MS analysis of the PET-dECM following trypsin digestion. Through a comprehensive analysis utilizing Proteome Discoverer 2.4 software for protein sequence identification, and by referencing amino acid sequence information for proteins like collagen from the UniProt protein database, Table 3 was formulated. As evident from the table, the protein varieties encompassed within the dECM coating of the PET-dECM, as identified by HPLC-MS, comprise collagen types I, II, III, IV, V, VI, and XI, alongside decorin and fibronectin. These discoveries correspond with the outcomes attained via FTIR spectroscopy, which uncovered distinct absorption peaks, signifying the existence of collagen and polysaccharides on the exterior of the PET-dECM specimens.

### 3.3. Biological Performance Test

#### 3.3.1. Observation of Cell Morphology

**Figure 3:**
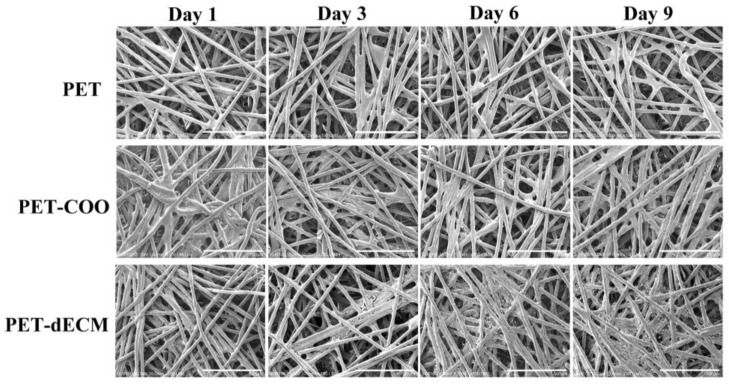
SEM photographs of HepaRG cells growing on PET, PET-COO, and PET-dECM scaffolds, taken on days 1, 3, 6, and 9. Scale bar: 500 μm.

The growth of HepaRG cells on the PET, PET-COO, and PET-dECM scaffolds was observed through a cold-field emission scanning electron microscope (Figure 3). Our research findings reveal that on the very first day of culturing, the cells began adhering to and accumulating on the surfaces of both the PET-COO and PET-dECM fibers. As the cultivation process continued, the population of the cells on the PET-dECM fibers saw a notable surge, with distinct clusters of cells forming by the 6th day. By the 9th day, a three-dimensional tissue structure, comprising extensively connected cell layers, had emerged. The PET-COO also facilitated some cell adhesion and growth, but it did not exhibit significant cell clustering as the cultivation progressed. In stark contrast, the untreated PET fibers only hosted a scant number of cells. Hydrophilicity stands as a pivotal criterion in evaluating a material’s biocompatibility, and our observations may be attributed to the material’s hydrophilic properties [46,47]. The PET-dECM, adorned with a dECM coating, showcases remarkable biocompatibility, fostering a conducive environment for cell growth and bolstering cell adhesion and proliferation.

#### 3.3.2. Cell Activity and Proliferation

**Figure 4:**
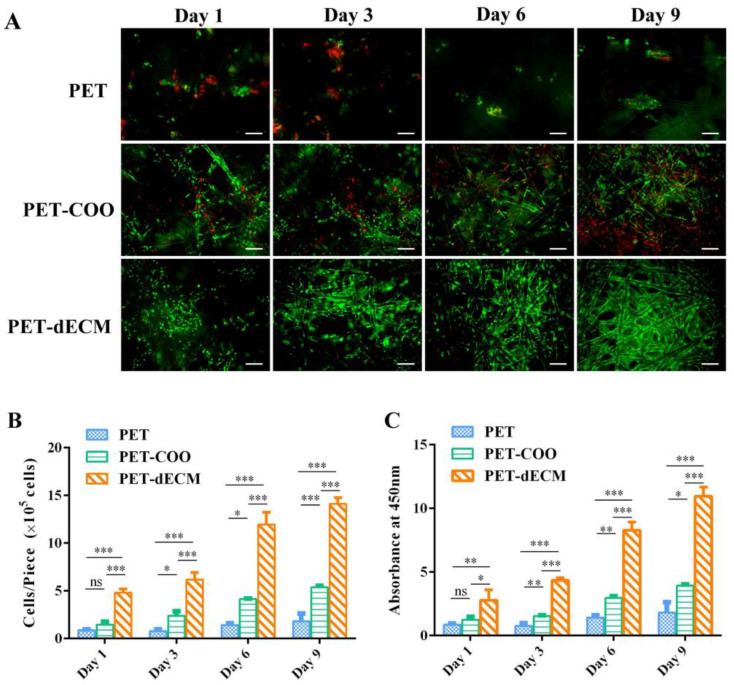
The activity and proliferation assay of the HepaRG cells cultivated on the PET, PET-COO, and PET-dECM scaffolds were conducted on days 1, 3, 6, and 9. (**A**) The live/dead fluorescence staining. The green indicates living cells and red indicates dead cells. Scale bar: 100 μm. (**B**) The cell counting results. (**C**) The CCK-8 results. The data are given as the mean ± SD, *n* = 3; *: *p* < 0.05, **: *p* < 0.01, ***: *p* < 0.001, and ns: not significant.

The principle of fluorescent staining for cell viability is based on the differential staining of live and dead cells using fluorescent dyes. This method allows researchers to visually distinguish between viable and non-viable cells under a fluorescent microscope. As the number of culture days increases, the PET-dECM scaffold gradually exhibits stronger green fluorescent signals, while no significant red fluorescent signals are observed (Figure 4A). This trend suggests that the PET-dECM demonstrates good biocompatibility and can efficiently promote cell growth and proliferation. Although green fluorescence is visible on the PET-COO scaffold, its intensity is notably weaker compared to that of the PET-dECM, and there is a conspicuous presence of red fluorescence. This indicates that the hepatocytes cultured on the PET-COO scaffold exhibit relatively low activity and a higher mortality rate. On the unmodified PET scaffold, predominantly red fluorescent signals are detected, with little to no green fluorescence. This finding aligns with the cell count trend observed through scanning electron microscopy in Section 3.3.1. Our results imply that the PET-dECM offers an ideal growth environment for HepaRG cells, fostering cell adhesion and proliferation. Conversely, the chemically inert interface of unmodified PET makes it unsuitable for cell culture, as it fails to adequately support cell growth and proliferation. To assess the effect of the PET, PET-COO, and PET-dECM scaffolds on HepaRG cell proliferation and viability, cell counting and CCK-8 assays were conducted on culture days 1, 3, 6, and 9. The results showed that as the culture progressed, the cell count on the PET-dECM scaffolds steadily increased (Figure 2B), mirrored by a proportional increase in OD450 nm absorbance (Figure 2C). By the ninth day of culture, the number of RG cells adherent to the PET-dECM reached 1.41 ± 0.52° × 10^6^. In comparison, when the same number of cells were inoculated, the PET and PET-COO scaffolds showed notably lower values for both metrics in contrast to the PET-dECM, and these differences were statistically significant. These findings imply that the dECM modification more effectively promotes HepaRG cell proliferation and maintains cell viability.

#### 3.3.3. Cell Polarity, Stemness, and Function

**Figure 5:**
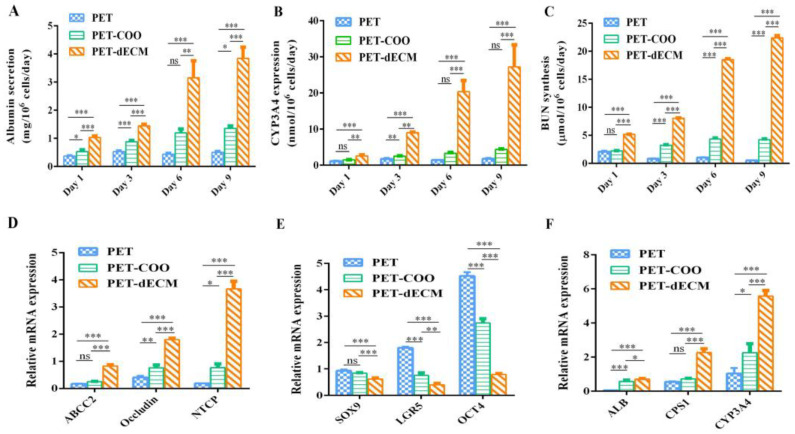
mRNA and protein expression level of HepaRG cells cultivated on PET, PET-COO, and PET-dECM scaffolds. (**A**) Albumin (ALB) secretion. (**B**) CYP3A4 expression. (**C**) BUN synthesis. (**D**) qRT-PCR analysis of *ABCC2*, *Occludin*, and *NTCP* levels related to polarity. (**E**) qRT-PCR analysis of *SOX9*, *LGR5*, and *OCT4* levels related to stemness. (**F**) qRT-PCR analysis of *ALB*, *CPS1*, and *CYP3A4* levels related to function. Data are given as mean ± SD, *n* = 3; *: *p* < 0.05, **: *p* < 0.01, ***: *p* < 0.001, and ns: not significant.

HepaRG cells are currently recognized as a biologically relevant surrogate model, widely acknowledged for bearing numerous traits of primary human hepatocytes. This cell model closely mirrors primary human hepatocytes in aspects such as morphology, the expression of crucial metabolic enzymes, nuclear receptor expression, and drug transporter functions. As an example, these cells display an ability to convert ammonia and synthesize urea, and they also exhibit high levels of drug-metabolizing enzymes. Notably, CYP3A4, CYP2B6, and albumin (ALB) match the expression levels found in primary human hepatocytes [48,49]. HepaRG cells have the capacity to differentiate into hepatocytes at a high density, making them a preferred cell source in the areas of liver tissue engineering and bioartificial liver research [50,51]. To comprehensively assess the functional capabilities of HepaRG cells cultured on PET-COO, PET-dECM, and PET scaffolds, we performed ELISA tests to measure the functional proteins and evaluate urea synthesis (Figure 5). Additionally, to eliminate the influence of varying cell quantities on different materials, we calibrated the cell count and evaluated the cellular function on a per-cell basis. The results indicated that as the culture period progressed, the albumin secretion level from cells grown on the PET-dECM scaffold steadily rose. Specifically, on days 1, 3, 6, and 9 of our measurements, the albumin secretion from the cells on the PET-dECM scaffold was notably higher compared to the cells grown on the PET and PET-COO scaffolds (*p* < 0.05) (Figure 5A). Mirroring this trend, the expression of CYP3A4 by cells cultivated on the PET-dECM scaffold also showed a gradual increase over time. On days 3, 6, and 9, the CYP3A4 expression on the PET-dECM scaffold surpassed that on the PET and PET-COO scaffolds significantly (*p* < 0.05) (Figure 5B). Additionally, we observed that while the albumin secretion and CYP3A4 expression of cells on the PET-COO scaffold were considerably lower than those on the PET-dECM, they were still significantly higher than those on the PET. This enhancement could be attributed to the augmented hydrophilicity and surface active groups of the material, resulting from the sodium hydroxide modification treatment.

Hepatic encephalopathy is a severe complication in patients with liver failure, primarily caused by ammonia poisoning. Under normal circumstances, liver cells convert ammonia in the body into urea through biochemical reactions, such as the ornithine cycle. The resulting urea is then excreted through the kidneys, helping to maintain ammonia balance in the body. However, when liver function is impaired, the ammonia conversion capability may decline. This can lead to ammonia accumulation in the body, subsequently inducing hepatic encephalopathy [52]. Urea, as the primary product of ammonia conversion, serves as a direct indicator of the ammonia conversion function of liver cells, reflected through its synthesis level.

To eliminate the influence of cell quantity, our experiment evaluated the urea synthesis capability of liver cells on three scaffolds: PET, PET-COO, and PET-dECM (Figure 5C). This was achieved by calibrating the cell count. The results revealed that as the culture days progressed, the urea nitrogen synthesis level per cell on the PET-dECM scaffold showed a gradual increase. Specifically, on days 1, 3, 6, and 9 of our measurements, the urea nitrogen synthesis level on the PET-dECM scaffold was significantly higher compared to the cells cultured on the PET and PET-COO scaffolds (*p* < 0.001). This finding indicates that the PET-dECM effectively supports the ammonia conversion function of liver cells.

Like other epithelial cells, hepatocytes possess the unique polarity of epithelial cells when cultured *in vitro* or *in vivo*. The unique physiological function of the liver is closely related to the polarity of hepatocytes, such as bile secretion and the synthesis and release of various proteins (such as albumin, lipoproteins, *etc*.) in serum, all highly dependent on the polar structure of hepatocytes [53]. When mutations occur in tight junction proteins within hepatocytes, it may hinder the formation of bile canaliculi, cause an abnormal aggregation of epithelial cells, and lead to disorders in the liver structure [54]. Therefore, when culturing hepatocytes *in vitro*, restoring and maintaining their polarity is crucial for enhancing their functional activity. Increasing evidence suggests that the establishment and maintenance of hepatocyte polarity are closely related to intercellular interactions and the interaction between cells and the extracellular matrix [55]. Based on HepRG cells exhibiting higher albumin secretion, CYP3A4 expression, and ammonia metabolism on the PET-dECM scaffold, this section examines the mRNA expression of polarity genes, stemness genes, and genes related to the aforementioned hepatocyte functions in HepRG cells cultured on different scaffolds on the sixth day. This exploration aims to understand the relationship between the polarity genes, stemness genes, and functional genes, thereby analyzing the potential mechanisms by which the PET-dECM enhances cellular functions. Hepatocyte polarity comprises three functional areas: the basal membrane area, lateral membrane area, and apical membrane area. Among them, NTCP is located in the basal membrane area, Occludin in the lateral membrane area, and ABCC2 in the apical membrane area. These proteins play a crucial role in maintaining and regulating the normal physiological functions of the liver. The results in Figure 5D show that compared to PET and PET-COO, the gene expression of *ABCC2*, *Occludin*, and *NTCP* in HepaRG cells cultured on the PET-dECM scaffold exhibits an upregulated trend. This suggests that the modification of PET material by the dECM promotes the polarity recovery of HepaRG cells cultured *in vitro* to a certain extent.

HepaRG, a type of human hepatic progenitor cell, possesses bidirectional differentiation potential and can differentiate into mature hepatocytes or cholangiocytes. *SOX9*, *LGR5*, and *OCT4* serve as stemness marker genes, reflecting the differentiation status of cells and determining the fate and stemness maintenance of hepatocytes. The results in Figure 5E show that compared to PET and PET-COO, the stemness genes *SOX9*, *LGR5*, and *OCT4* are downregulated in HepaRG cells cultured on the PET-dECM scaffold. These findings suggest, to some extent, that the PET-dECM can promote the differentiation of HepaRG cells rather than maintain their stemness state.

Figure 5F further examines the mRNA expression of *ALB*, *CYP3A4*, and *CPS1*, a key rate-limiting enzyme in the urea cycle. The results indicate that compared to PET and PET-COO, these functional genes characterizing hepatocytes show an upregulated trend, consistent with the protein expression patterns.

Through analyzing the expression results of the polarity genes, stemness genes, and functional genes, it is evident that the enhanced hepatocyte function of HepaRG on the PET-dECM may be related to the dECM’s ability to promote cell polarity recovery. During this process of cell polarity restoration, the stemness of HepRG cells decreased, inducing their differentiation toward a hepatic lineage.

## 4. Conclusions

This study utilizes a natural human umbilical cord-derived extracellular matrix as a modifying agent for PET, aiming to synthesize a dECM-coated polyester fiber-based scaffold suitable for liver tissue engineering applications. We successfully prepared a decellularized human umbilical cord-derived ECM by employing a combined decellularization strategy involving trypsin/EDTA, TritonX-100, and deoxycholate. Through the carboxylation of PET materials, EDC/NHS activated crosslinking, dECM coupling, and additional steps, a novel PET-dECM material was crafted. Furthermore, this article thoroughly explores and characterizes the physicochemical properties, biological performance, and mechanism of the material. The ultimate objective is to lay a foundation and prepare the ground for its potential future applications in the biomedical field. The results obtained reveal the following findings:

- The XPS analysis revealed that the N1s spectrum of the PET-dECM shows binding energies at 399.8 eV, corresponding to the amide groups formed after dECM modification. Concurrently, the N element content on the material surface amounted to 8.95%, suggesting that the surface element composition has changed after the modification treatment. The PET-dECM displays characteristic absorption peaks of collagen, specifically Amid II at 1554 cm^−1^, Amid I at 1713 cm^−1^, Amid B at 2916 cm^−1^, and Amid A at 3307 cm^−1^. Additionally, a characteristic absorption peak indicating the presence of carbohydrates is observed at 1042 cm^−1^. The dECM coating significantly enhanced the hydrophilicity of the PET material, resulting in a decrease in the water contact angle of the PET-dECM scaffold from 132.97° to 67.71° (*p* < 0.001). The HPLC-MS analysis of the PET-dECM revealed that the dECM coating on PET comprises collagen types I, II, III, IV, V, VI, and XI, alongside decorin and fibronectin.
- Based on the results obtained from electron microscopy experiments, cell viability staining, cell counting, and CCK-8 cell activity assays, it was evident that the PET-dECM material facilitated the adhesion and proliferation and maintained the activity of HepaRG cells. Furthermore, the PET-dECM significantly surpassed PET and PET-COO in terms of albumin secretion, urea synthesis, and CYP3A4 expression. Additional analysis of the transcription levels of genes associated with the polarity, stemness, and function of HepaRG cells cultured on different materials showed that the RNA transcription levels of *ABCC2*, *Occludin*, and *NTCP* were considerably upregulated on the PET-dECM, while *SOX9*, *LGR5*, and *OCT4* were significantly downregulated. *ALB*, *CYP3A4*, and *CPS1* demonstrated an upward transcriptional trend. These findings imply that PET-dECM material may regulate and induce differentiation by aiding in the restoration of HepaRG cell polarity.

## Figures and Tables

**Table 1:** Primers for qRT-PCR.

Gene	Forward Primer (5′-3′)	Reverse Primer (5′-3′)
*ABCC2*	*TCTCTCGATACTCTGTGGCAC*	*CTGGAATCCGTAGGAGATGAAGA*
*Occludin*	*GACTTCAGGCAGCCTCGTTAC*	*GCCAGTTGTGTAGTCTGTCTCA*
*NTCP*	*GGCCGTCACAGTTCTCTCTG*	*GGTGGCAATCAAGAGTGGTGT*
*SOX9*	*AGCGAACGCACATCAAGAC*	*CTGTAGGCGATCTGTTGGGG*
*LGR5*	*CACCTCCTACCTAGACCTCAGT*	*CGCAAGACGTAACTCCTCCAG*
*OCT4*	*GGGAGATTGATAACTGGTGTGTT*	*GTGTATATCCCAGGGTGATCCTC*
*ALB*	*TGCAACTCTTCGTGAAACCTATG*	*ACATCAACCTCTGGTCTCACC*
*CPS1*	*AATGAGGTGGGCTTAAAGCAAG*	*AGTTCCACTCCACAGTTCAGA*
*CYP3A4*	*AAGTCGCCTCGAAGATACACA*	*AAGGAGAGAACACTGCTCGTG*
*β-Actin*	*CATGTACGTTGCTATCCAGGC*	*CTCCTTAATGTCACGCACGAT*

**Table 2:** Surface elementary composition of the scaffolds from XPS analysis.

Sample	Elementary Composition		
	C (%)	N (%)	O (%)
PET	71.34	0.47	27.91
PET-dECM	67.03	8.95	23.53

**Table 3:** Types of proteins in PET-dECM coating by HPLC-MS analysis.

Protein Sequence Number	Protein Type	Coverage Rate (%)	Peptides	Unique Peptides	Molecular Weight (kDa)
P02452	Collagen alpha-1(I) chain	26	27	5	138.9
A0A384MDU2	Collagen, type I, alpha 2	30	29	17	129.2
P28481	Collagen alpha-1(II) chain	3	3	1	141.9
P02461	Collagen alpha-1(III) chain	24	26	25	138.5
A0A024RDW8	Collagen, type IV, alpha 2	4	5	5	167.4
B2ZZ86	Collagen type V alpha 1	6	8	7	183.5
P05997	Collagen alpha-2(V) chain	10	10	10	144.8
D9ZGF2	Collagen, type VI, alpha 3	4	12	12	343.5
D3DT71	Collagen, type XI, alpha 1	2	3	2	176.5
P07585	Decorin	3	1	1	39.7
A0A024R462	Fibronectin	3	4	4	259

## Data Availability

The data presented in this study are available on request from the corresponding author. The data are not publicly available due to privacy.
